# Supplementary material for: Acoustic spin-Chern insulator induced by synthetic spin–orbit coupling with spin conservation breaking
Source: Nat Commun. 2020 Jun 26;11:3227. doi: 10.1038/s41467-020-17039-1 (PMC7320166; doi:10.1038/s41467-020-17039-1)
Supplement: Supplementary file 1 — Supplementary Information [file 41467_2020_17039_MOESM1_ESM.pdf]

Supplementary Information for

Acoustic spin-Chern insulator induced by synthetic spin-orbit coupling with spin conservation breaking

Deng et al.

Supplementary Figures 1 to 9

Supplementary Notes 1 to 10

Supplementary References

Supplementary Figures

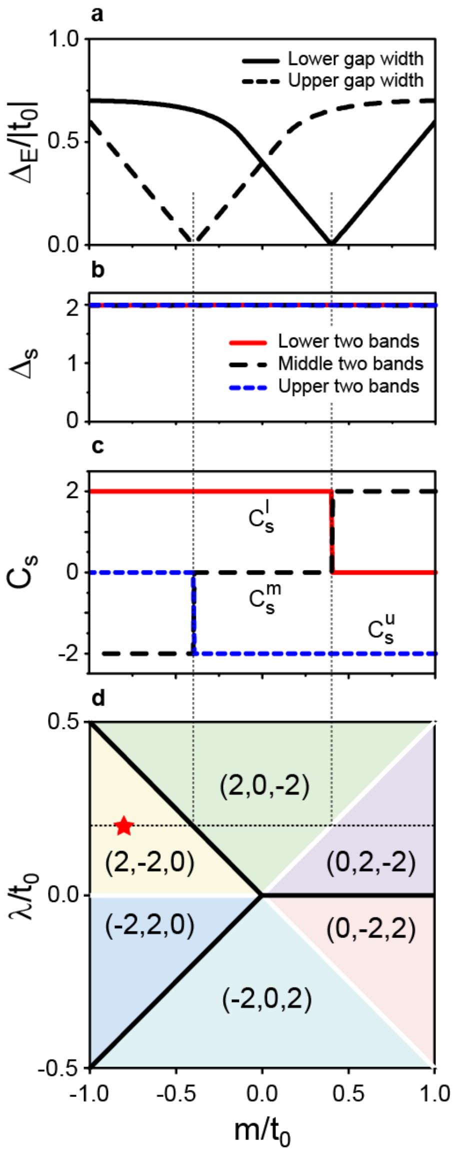

Supplementary Figure 1 | Bulk and spin gaps and phase diagram determined by the spin-Chern numbers. a, The bulk band gaps. The lower gap is the gap between the second and third bands, while the upper gap is that between the fourth and fifth bands. **b,** The spin spectrum gaps. **c,** The spin-Chern numbers $C_s = (C_s^l, C_s^m, C_s^u)$ of the lower, middle, upper two bands, as function of m/t_0. The interlayer coupling is chosen such that $\lambda = 0.2t_0$. **d,** Phase diagram determined by C_s in the λ/t_0 and m/t_0 plane. The white (black) line represents the lower (upper) bulk gap closure.

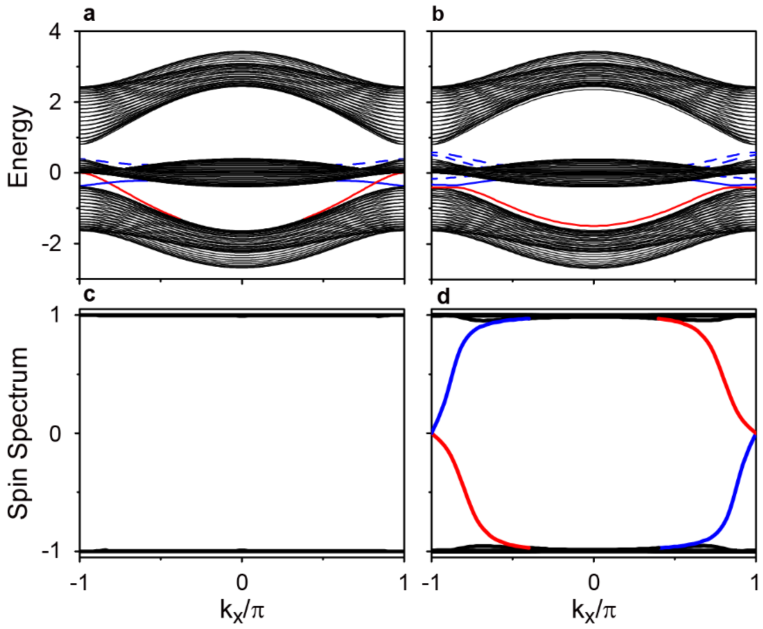

Supplementary Figure 2 | The projected energy and spin spectrums of ribbons of lattice model. a, and **c,** The parameters are the same as Fig. 1d of the Main Text. **b,** and **d,** The parameters are similar with **a** and **c**, but adding the edge potentials with the interlayer coupling between sites $B_\uparrow$ and $B_\downarrow$ on the boundaries $t_B = -0.5$. The energy gap or spin gap needs to close on the sample boundaries, because of the nonzero spin-Chern number.

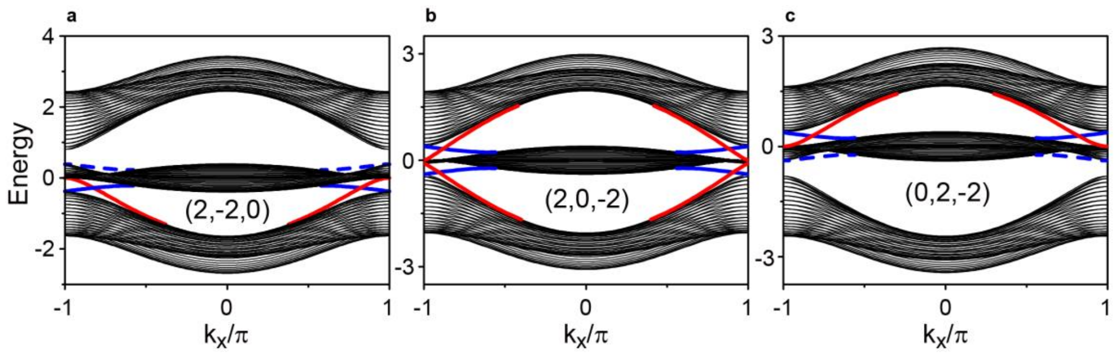

Supplementary Figure 3 | The projected dispersions of ribbons for other SCI phases. The parameters are **a,** $m = 0.8$, **b,** $m = -0.1$ and **c,** $m = -0.8$. The other parameters are the same as in Fig. 1 of the Main Text.

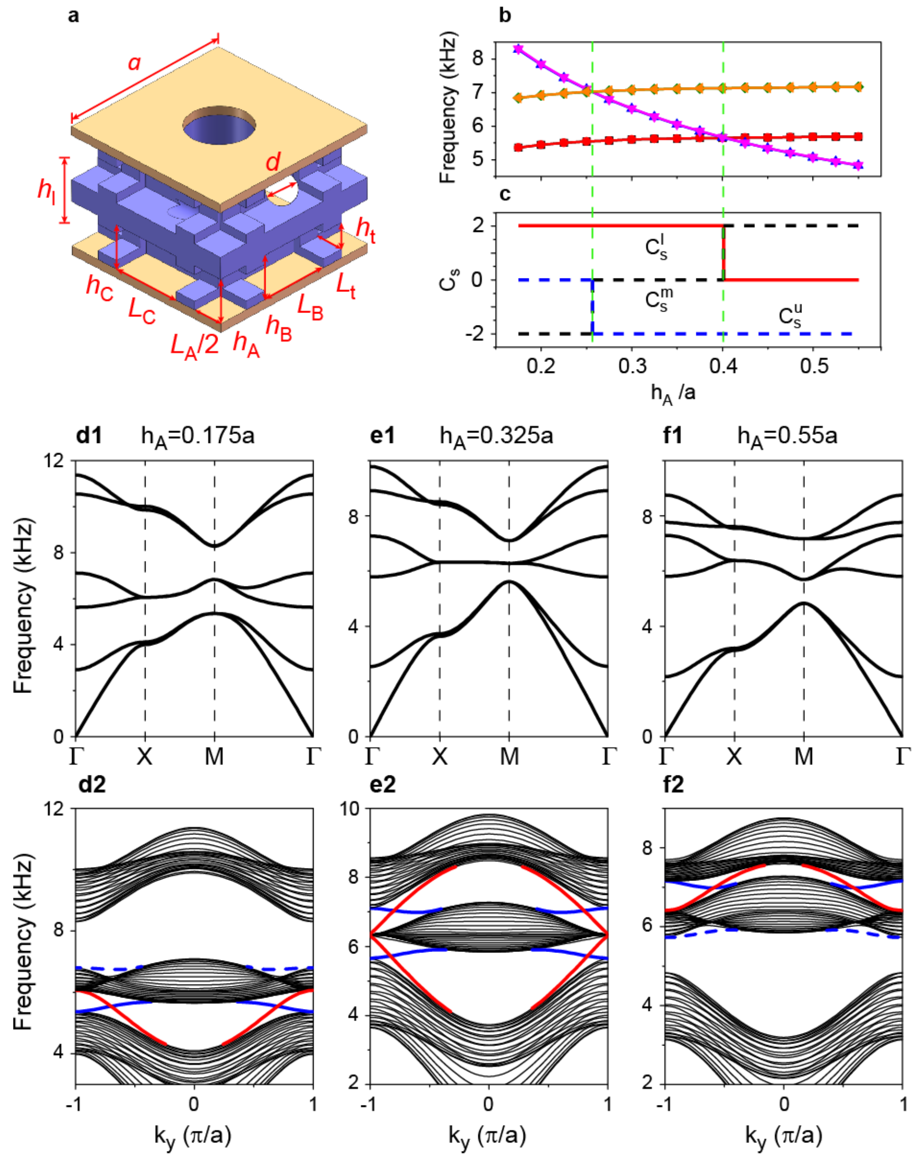

Supplementary Figure 4 | Band inversions and three topological phases of the ASCI in the PC. a, The unit cell of the PC with specific parameters. **b,** The bulk bands at M point as a function of the height of cavity A (h_{A}). Band inversions occur at $h_{\mathrm{A}} = 0.255a$ and $h_{\mathrm{A}} = 0.4a$. **c,** The corresponding spin-Chern numbers C_s of the PC. The topological phase transitions occur at the gap closing points. Bulk bands and projected band dispersions of ribbons in three topologically distinct regions of the phase diagram **d,** $h_{\mathrm{A}} = 0.175a$, **e,** $h_{\mathrm{A}} = 0.325a$ and **f,** $h_{\mathrm{A}} = 0.55a$.

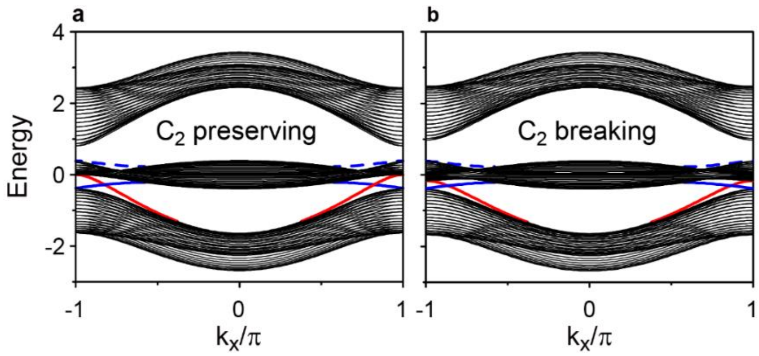

Supplementary Figure 5 | The projected dispersions of ribbons for the SCI systems with and without C_2 symmetry. The lattice model extends the one of Fig. 1a of the Main Text, in which the two nearest-neighbor intralayer hoppings along the x direction are $t_0 \pm dt_x$, and those along the y direction are still t_0. **a,** The system has C_2 symmetry with $dt_x = 0$. **b,** The system breaks C_2 symmetry with $dt_x = 0.2$. The other parameters are $t_0 = -1$, $\lambda = -0.2$, and $m = 0.8$. The red and blue lines represent the boundary states localize at two different boundaries of the ribbon.

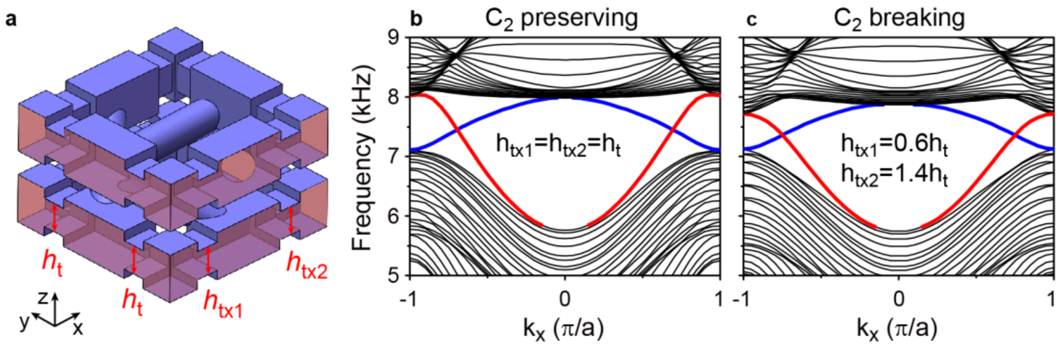

Supplementary Figure 6 | The projected dispersions of ribbons for the SCI systems with and without C2 symmetry in the PC. a, The extended unit cell of the PC shown in Fig. 2b of the Main Text, where the heights of two intralayer tubes along the x direction are h_{tx1} and h_{tx2}, respectively. **b,** The PC has C2 symmetry with $h_{tx1} = h_{tx2} = h_t$. **c,** The PC breaks C2 symmetry with $h_{tx1} = 0.6h_t$ and $h_{tx2} = 1.4h_t$. The gapless helical boundary states exist in both these two acoustic ribbons, independent on the C2 symmetry.

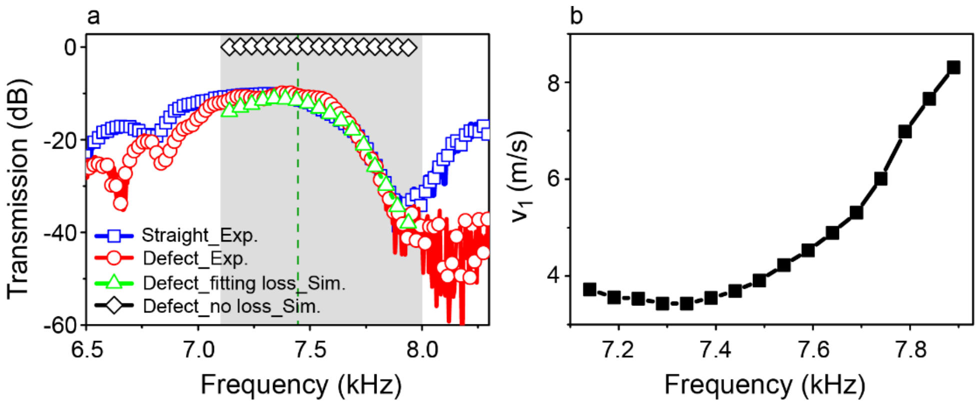

Supplementary Figure 7 | The influence of air loss on the transmission. a, The simulated with (triangle) and without (diamond) loss, and measured (circle) transmissions for the defect path, and the measured transmission (square) for a sample with a straight path having the same length. **b,** The fitting loss v_1 as a function of frequency in the bulk gap.

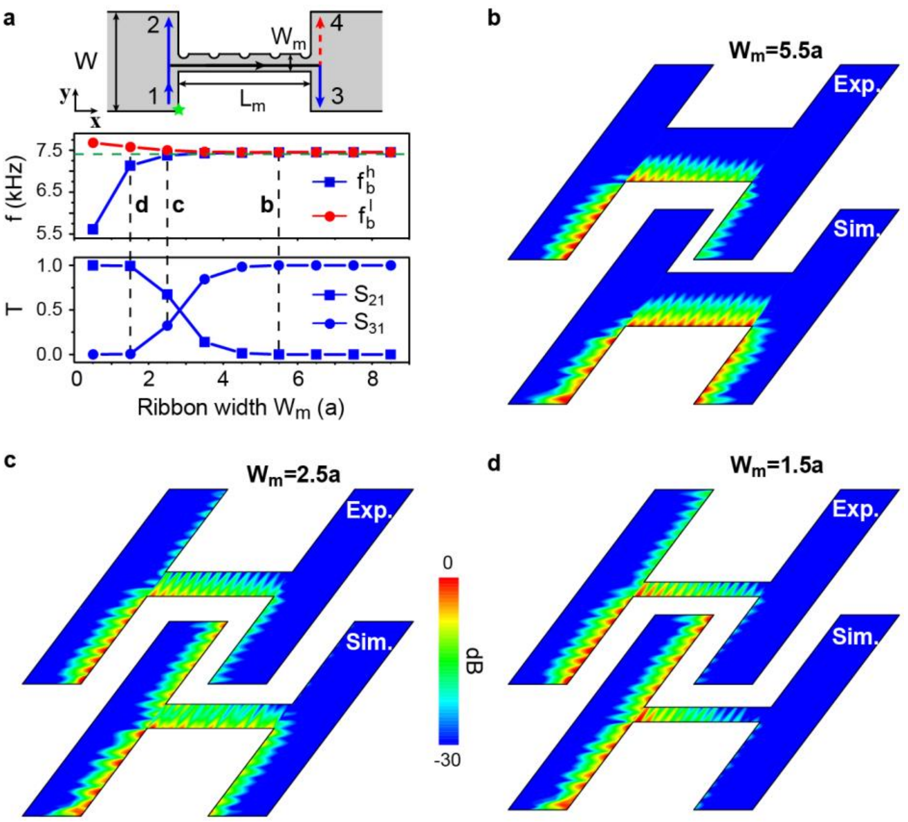

Supplementary Figure 8 | The partition behavior of the acoustic boundary waves in an H-shaped ASCI. a, Top panel: the schematic of the H-shaped PC, where the green star denotes the position of the source. Middle panel: the lowest (highest) frequency f_{b}^{l} (f_{b}^{h}) of the upstream (downstream) branch of the boundary states in the lower gap as a function of the width of the middle ribbon W_{m}. The dashed green line denotes the excitation frequency of 7.44 kHz. Bottom panel: the calculated transmission from channel 1 to terminals 2 (S_{21}) and 3 (S_{31}). **b-d,** The simulated and experimental field distributions in the H-shaped PC for three different W_{m} (the dashed black lines in **a**). The boundary waves can be controlled by tuning W_{m}.

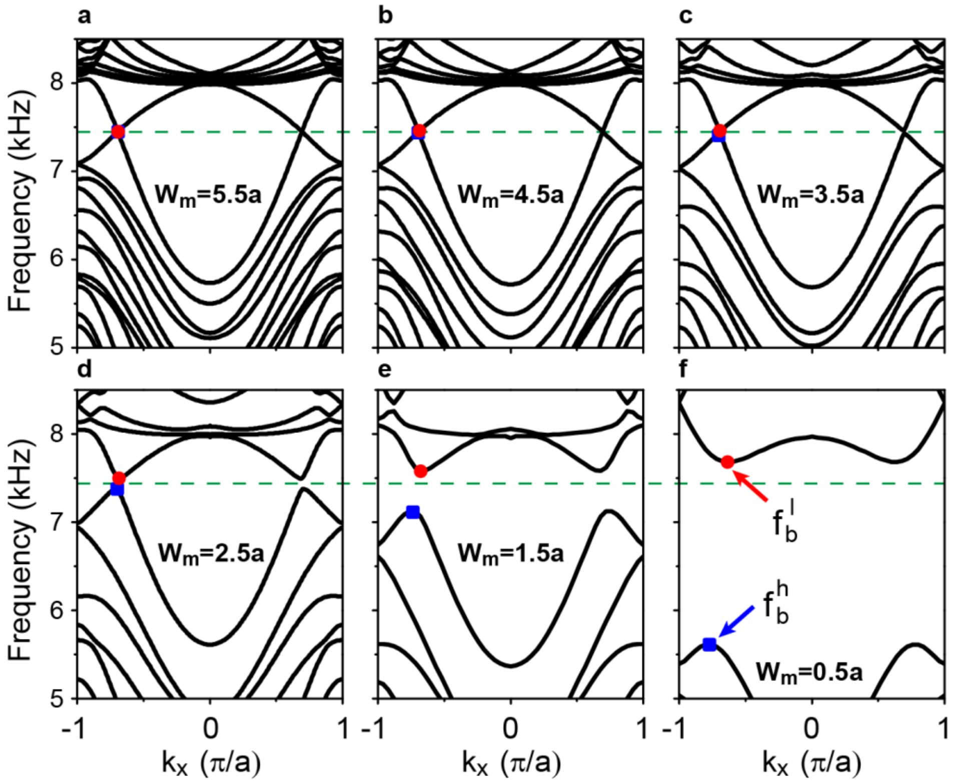

Supplementary Figure 9 | The projected dispersions of the middle channel in the H-shaped PC for different widths. The width W_m is changed from wide to narrow as **a,** $W_\mathrm{m} = 5.5a$, **b,** $W_\mathrm{m} = 4.5a$, **c,** $W_\mathrm{m} = 3.5a$, **d,** $W_\mathrm{m} = 2.5a$, **e,** $W_\mathrm{m} = 1.5a$ and **f,** $W_\mathrm{m} = 0.5a$.

Supplementary Note 1. The synthetic spin-orbit interaction

In this section, we derive the Hamiltonian in momentum space, and show how the interlayer coupling induces the synthetic spin-orbit interaction and breaks the pseudospin conservation. Taking the basis $(A_\uparrow, B_\uparrow, C_\uparrow, A_\downarrow, B_\downarrow, C_\downarrow)^T$, where $\uparrow$ ($\downarrow$) denotes the pseudospin of upper (lower) layer, and taking the Fourier transformation of Hamiltonian Eq. (1) in the Main Text, the Bloch Hamiltonian is given by

$$H_k = \begin{pmatrix} m & t_x & t_y & 0 & 0 & 0 \\ t_x^* & 0 & 0 & 0 & 0 & t_- \\ t_y & 0 & 0 & 0 & t_+ & 0 \\ 0 & 0 & 0 & m & t_x & t_y \\ 0 & 0 & t_+ & t_x^* & 0 & 0 \\ 0 & t_- & 0 & t_y & 0 & 0 \end{pmatrix}, \quad (1)$$

where $t_x = 2t_1 \cos(k_x/2) + 2it_\delta \sin(k_x/2)$, $t_y = 2t_0 \cos(k_{x,y}/2)$ and $t_\pm = 2\lambda \cos[(k_x \pm k_y)/2]$, the lattice constant is set to unity. By applying a unitary transformation $H_u = UH_kU^\dagger$, where

$$U = \frac{1}{\sqrt{2}}\begin{pmatrix} 1 & -i \\ 1 & i \end{pmatrix} \otimes I_3, \quad (2)$$

with I_3 the unit matrix of size 3x3, the Hamiltonian is transformed into

$$H_u = \begin{pmatrix} m & t_x & t_y & 0 & 0 & 0 \\ t_x^* & 0 & it_s & 0 & 0 & -it_c \\ t_y & -it_s & 0 & 0 & -it_c & 0 \\ 0 & 0 & 0 & m & t_x & t_y \\ 0 & 0 & it_c & t_x^* & 0 & -it_s \\ 0 & it_c & 0 & t_y & it_s & 0 \end{pmatrix}, \quad (3)$$

with

$$t_s = (t_- - t_+)/2 = 2\lambda \sin(k_x/2) \sin(k_y/2), \quad (4)$$

$$t_c = (t_- + t_+)/2 = 2\lambda \cos(k_x/2) \cos(k_y/2). \quad (5)$$

The t_s term has the same form of the intrinsic spin-orbit interaction with strength $\lambda/2$ [1], whereas the t_c term breaks the spin conservation. The Hamiltonian H_u is the spin-Chern insulator (SCI) Hamiltonian, which can be understood as follows: when t_c term vanishes, the Hamiltonian H_u is the SCI Hamiltonian with spin conservation. In this case, the t_s term opens a bulk gap and results in nontrivial band topology of SCI phase that can be captured in terms of Chern numbers given that the spin is conserved.

Supplementary Note 2. Spin-Chern numbers

In this section, we calculate the bulk topological invariant, namely, the spin-Chern numbers, to characterize the SCI phases of the lattice model.

This system cannot be described by the Z_2 index associated with the spin-1/2 time-reversal symmetry. The spin-1/2 time-reversal operator is given as $T = i\sigma_y K \otimes I_3$ with $T^2 = -1$, where σ_y acts on the layer pseudospin and K is the complex conjugation. For the Hamiltonian H_u, $[H_u, T] = 0$ at the first Brillouin zone boundaries but generically $[H_u, T] \neq 0$ for generic momenta. This observation explains the Kramers-like double degeneracy of the tight-binding model bands at the Brillouin zone boundaries. However, the absence of an antiunitary symmetry for the full system prevents us from using the Z_2 index associated with the spin-1/2 time-reversal symmetry [2].

The spin-Chern numbers have originally been introduced to describe Z_2 topological insulators in the presence of time-reversal symmetry. In fact, in the presence of both time reversal symmetry and spin conservation, the spin-Chern number and the Z_2 index give rise to the same topological classification [3-5]. Since the definition of spin-Chern numbers does not rely on any symmetries, it can be extended to pseudospin systems by replacing the spin operator by the pseudospin operator. Therefore, the spin-Chern numbers can be used to study topological insulators for classical waves, where the time-reversal symmetry is bosonic, i.e., it acts as complex conjugation, and essentially different from that of spin-1/2 systems.

The spin-Chern numbers C_s can be calculated without spin conservation in a standard way, as long as the energy band gap and spin spectrum gap stay open. As an example, we first calculate the spin-Chern number C_s^l for the lower two bands of Hamiltonian H_k. For a given λ, the energy band gap between the second and third bands, namely the lower band gap, is shown as the black solid line in Supplementary Fig. 1a. The lower band gap first decreases, then closes at $m = 2\lambda$, and reopens with increasing m. The second necessary condition for defining spin-Chern numbers is the presence of spin spectrum gap. The spin operator of the original Hamiltonian H_k is

considered as $\tau_y = U^\dagger(\sigma_z \otimes I_3)U = \sigma_y \otimes I_3$, since the spin operator of H_u is $\sigma_z \otimes I_3$. The Hamiltonian H_s^l projected into the spin space is

$$H_s^l(\boldsymbol{k}) = \begin{pmatrix} \langle \varphi_1 | \tau_y | \varphi_1 \rangle & \langle \varphi_1 | \tau_y | \varphi_2 \rangle \\ \langle \varphi_2 | \tau_y | \varphi_1 \rangle & \langle \varphi_2 | \tau_y | \varphi_2 \rangle \end{pmatrix}, \tag{6}$$

where the bases $\varphi_1(\boldsymbol{k})$ and $\varphi_2(\boldsymbol{k})$ are the Bloch wavefunctions of the lower two bands. Diagonalizing H_s^l, one obtains two spin bands $\chi_\pm^l(\boldsymbol{k})$ and the related eigenvectors $\phi_\pm^l(\boldsymbol{k}) = (\alpha_\pm, \beta_\pm)^T$. The spin spectrum gap of the lower two bands is defined as $\Delta_s^l = \min|\chi_+^l(\boldsymbol{k}) - \chi_-^l(\boldsymbol{k})|$, which is plotted as the red solid line in Supplementary Fig. 1b. One can see that the spin spectrum gap is always open with varying m. With an open spin gap, the wavefunctions of the lower two bands can be projected into two spin sectors, as

$$\psi_\pm^l(\boldsymbol{k}) = \alpha_\pm(\boldsymbol{k})\varphi_1(\boldsymbol{k}) + \beta_\pm(\boldsymbol{k})\varphi_2(\boldsymbol{k}). \tag{7}$$

One can define a Chern number for each spin sector as

$$C_\pm^l = \frac{1}{2\pi}\int d^2k\Omega_\pm^l(\boldsymbol{k}), \tag{8}$$

where $\Omega_\pm^l = \hat{e}_z \cdot \left[\nabla_k \times \langle \psi_\pm^l(\boldsymbol{k}) | i\nabla_k | \psi_\pm^l(\boldsymbol{k}) \rangle\right]$ are the spin-projected Berry curvatures. The spin-Chern number C_s^l is defined as $C_s^l = C_+^l - C_-^l$, which is calculated and shown as the red solid line in Supplementary Fig. 1c. We find that $C_s^l = 2$ for $m < 2\lambda$, and $C_s^l = 0$ for $m > 2\lambda$. The phase boundary corresponds to a closing of the energetic band gap at $m = 2\lambda$ (the right green dotted line). The spin-Chern numbers of the middle two bands C_s^m and upper two bands C_s^u can be calculated with the same procedure. The bulk band topology of the system can be entirely characterized by $C_s = (C_s^l, C_s^m, C_s^u)$. A rich topological phase diagram determined by C_s in the λ/t_0 and m/t_0 plane is shown in Supplementary Fig. 1d. There are six topologically distinct phases. At the boundaries among them, the energetic band gap closes. The white and black lines represent the lower and upper gap closing transition, respectively.

Supplementary Note 3. The generalized bulk-boundary correspondence in the SCI

The nonzero spin-Chern number guarantees that the existence of the helical

boundary states in the bulk gap, but whether gapless or not, depends on the system symmetry and micro-structure of the sample boundary [6]. If the spin gap remains open at the edges of the sample (Supplementary Fig. 2c), the energy gap has to close due to the non-trivial bulk topology captured by the spin-Chern number. As shown in Supplementary Fig. 2a, robust topology-protected gapless edge modes appear at the sample's boundaries. On the other hand, when the boundary states are gapped by adding edge potentials (Supplementary Fig. 2b), their spin spectrum must be gapless, as shown in Supplementary Fig. 2d. The gapped boundary states display the unusual pattern of the spin spectrum, originating from the nontrivial band topology. These two connections of boundary states to the bulk topological invariance, i.e. spin-Chern number, can be viewed as a generalized bulk-boundary correspondence. The stable existence of the boundary states is a desirable feature for practical applications of topological insulators.

Supplementary Note 4. Boundary states of six topologically distinct SCI phases

In this section, we calculate the boundary states of the SCI in six topologically distinct phases shown in Supplementary Fig. 1d. For the phase with $C_s = (2, -2, 0)$, the projected band dispersion for a ribbon is plotted in Supplementary Fig. 3a. The boundary states in the lower gap have been shown in Fig. 1d of the Main Text, corresponding to $C_s^l = 2$. The boundary states in the upper gap are only localized at the middle bands (dashed blue lines), indicating the trivial topology of the lower four bands with $C_s^l + C_s^m = 0$. For the phase with $C_s = (2, 0, -2)$, the related boundary state dispersion for a ribbon is presented in Supplementary Fig. 3b. There are helical boundary states both in the lower and upper gaps. Again, the red lines represent the boundary states localize at one boundary of the ribbon, while the blue lines stand for the boundary states localize at the other boundary. In the lower gap, a pair of counter-propagating boundary states with opposite spin polarizations exists at each boundary, accordingly to $C_s^l = 2$. The configuration of spin-momentum locking of boundary states in the upper gap is the same to the lower one, because of the same topological properties. For the phase with $C_s = (0, 2, -2)$, the projected band dispersion of a

ribbon is shown in Supplementary Fig. 3c. There are no gapless boundary states in the lower gap, due to the trivial topological invariant $C_s^l = 0$, while there exist helical boundary states in the upper gap, consistent with the total spin-Chern number of the lower four bands $C_s^l + C_s^m = 2$. The other three SCI phases for $\lambda/m < 0$ have the same projected band dispersions of those for $\lambda/m > 0$ with opposite spin polarizations, because of the opposite spin-Chern numbers.

Supplementary Note 5. Band inversion and topological phase transition in the real phononic crystal

In the Main Text, we realize the ASCI phase with spin-Chern numbers $C_s^l = 2$ in a specific phononic crystal (PC). The PC is designed to optimize the size of the lower band gap to easily detect and manipulate the boundary states in the experiment. In this section, we simulate the bulk band dispersion and show the topological phase transition in the same PC of the Main Text with different parameters. The unit cell is shown in Supplementary Fig. 4a, where the parameters are chosen as $a = 20$ mm, $h_l = 12$ mm, $h_\text{B} = h_\text{C} = 4.5$ mm, $L_\text{B} = L_\text{C} = 7$ mm, $L_\text{A} = 8$ mm, $L_t = 2.1$ mm, $h_t = 3$ mm, $d = 2.1$ mm. Here, h_l is the distance between the centers of two layers. In Supplementary Fig. 4b, the frequencies at the M point are calculated as a function of the height of A cavity h_A, where each acoustic mode is represented by a different color. Increasing h_A corresponds to decreasing m, since the resonance frequency reduces by expanding the volume of the cavity. The gap among the middle and upper two bands closes at $h_\text{A} = 0.255a$ (the first dashed green line on the left) giving rise to a band inversion. The lower gap among closes at $h_\text{A} = 0.4a$ (the second dashed green line on the right). So changing h_A allows to transition between three topologically distinct phases of the ASCI. The respective C_s are shown in Supplementary Fig. 4c. The topological phase transitions occurs at the band inversion points.

As further evidences, we show in Supplementary Figs. 4d-4f the bulk dispersions and relevant projected dispersions of ribbons for the three different phases. For the region $h_\text{A} < 0.255a$ shown in Supplementary Figs. 4d1 and 4d2, helical boundary states appear only in the lower gap, corresponding to the spin-Chern numbers $C_s =$

$(2, -2{,}0)$. For the region $0.255a < h_{\mathrm{A}} < 0.4a$ plotted in Supplementary Figs. 4e1 and 4e2, both the lower and upper gaps have helical boundary states, corresponding to $C_s = (2{,}0, -2)$. The lower gap remains open, while a band inversion occurs between the middle and upper two bulk bands. For the region $h_{\mathrm{A}} > 0.4a$ shown in Supplementary Figs. 4f1 and 4f2, the helical boundary states exist in the upper gap with $C_s = (0{,}2, -2)$, since the lower gaps closes and reopens. These findings are consistent with the discrete lattice model shown in Fig. 1 of the Main Text. The other SCI phases for $\lambda/t_0 < 0$ are not easy to realize in the PC, since couplings of opposite signs are needed for the intralayer and interlayer hoppings.

Supplementary Note 6. The boundary state dispersions in the absence of crystalline symmetries

The spin-Chern number remains well-defined when the crystalline symmetries are broken, since its definition does not rely on any symmetries [3, 4]. The only requirement is that the disorder preserves simultaneously both an open energy and spin gap [6]. Here, as a concrete example, we calculate the boundary state dispersions when the C_2 symmetry is broken by considering two different hoppings along the x direction. Since the spin-Chern number is still the same to the one with the C_2 symmetry, the system in the absence of C_2 symmetry still hosts the gapless helical boundary states, as shown in Supplementary Fig. 5 of the tight-binding model and Supplementary Fig. 6 of the acoustic structure.

Supplementary Note 7. The influence of air loss on the transmission and topology

In the Main Text, we argue that the measured transmission below that of simulation is attributed to air loss in experiment. To support this point, we calculate the transmission with air loss, and compare it with the measured one. As the green triangles and red circles shown in Supplementary Fig. 7a, one can see that they match well with each other. Here, the loss in simulation is introduced by adding an imaginary part to the sound velocity, i.e. $v = v_0 + iv_1(f)$. As shown in Supplementary Fig. 7b, $v_1(f)$ are

obtained by simulating the measured transmissions through a straight path (blue square in Supplementary Fig. 7a).

In addition, the losses in the acoustic system or alike systems mainly come from the intrinsic and unavoidable absorption in air, which makes the systems non-Hermitian. However, the losses would not change the topology of the systems. With the loss, the wave equation of sound changes from $\nabla^2 p_0 = \frac{\omega_0^2}{v_0^2} p_0$ for the Hermitian system to $\nabla^2 p = \frac{\omega^2}{v^2} p$ for the non-Hermitian system, where p_0 or p denotes the pressure field. One can find that p_0 still satisfies the non-Hermitian equation, as long as $\frac{\omega^2}{v^2} = \frac{\omega_0^2}{v_0^2}$, which gives $\omega = \omega_0 + i\frac{v_1}{v_0}\omega_0$. The complex frequency here implies that the pressure field in non-Hermitian system is damping with time, described by the imaginary part originating from the loss indicated by v_1, while the frequency of oscillation, described by the real part remains the same as the Hermitian system. Since $p = p_0$, the non-Hermitian system has the same spin-Chern number defined in Eq. (S4), and hosts the same topology as the Hermitian counterpart.

Supplementary Note 8. A switch effect of the acoustic boundary waves in an H-shaped sample

In the electronic system, a switch effect of the topological boundary states by means of a quantum point contact has been predicted [7]. Here, we experimentally realize such a novel transport phenomenon for the ASCI boundary waves. Supplementary Figure 8a (top panel) shows a schematic of the H-shaped structure of the ASCI, where the width of the left and right ribbons is $W = 20a$, and the length of the middle ribbon ("bridge") is $L_{\mathrm{m}} = 13a$. In the middle panel of Supplementary Fig. 8a, we show the lowest (highest) frequency f_{b}^{l} (f_{b}^{h}) of the upstream (downstream) branch of the boundary states in the lower gap as a function of the width of the middle ribbon W_{m} (more details shown in S-IX). When W_{m} is sufficiently large, as shown in Fig. 3 of the Main Text, the boundary wave dispersions on the opposite boundaries are gapless due to the lack of interactions between them. Hence, $f_{\mathrm{b}}^{l} = f_{\mathrm{b}}^{h}$. By gradually

decreasing W_{m}, the overlap of the surface waves localized at opposite boundaries can open a gap in the dispersions of the edge states. This results in a frequency mismatch between f_{b}^{l} and f_{b}^{h}. We calculate the transmissions from channel 1 to terminals 2 and 3 at 7.44 kHz and show the results in the bottom panel of Supplementary Fig. 8a The boundary waves can cross the bridge and reach terminal 3 for $W_{\mathrm{m}} > 4a$ and are completely blocked from terminal 2 for $W_{\mathrm{m}} < 2a$. The crossover from one to zero for S_{31} (transmission between channel 1 and terminal 3) occurs sharply as a function of W_{m}, indicating ideal partition transport of the boundary waves in the H-shaped ASCI.

In Supplementary Figs. 8b-8d, the pressure distributions of the boundary waves are shown for three different W_{m} values from wide to narrow. The upper (lower) panel is the measured (calculated) result. For $W_{\mathrm{m}} = 5.5a$, the boundary states completely propagate to terminal 3 because of the gapless boundary wave dispersions. For $W_{\mathrm{m}} = 2.5a$, the boundary waves can partly propagate both to the right and back to the left ribbon. For $W_{\mathrm{m}} = 1.5a$, the boundary waves are mainly blocked to terminal 2 since the finite size effect opens a large gap in the boundary wave dispersions. Due to air losses, the measured transmission of the boundary waves cannot reach unity, as in the simulated results in the absence of dissipations. However, the partition behavior shows good agreement between the simulations and the experiment. These results provide an efficient way to control the boundary waves.

The device based on the H-shaped structure in Supplementary Fig. 8 is designed to serve as a splitter. The principle behind is the coupling of the helical boundary states on the inner edges of the H-shaped structure, abiding by the corresponding spin-momentum locking. The width and length of the bridge bar in ‘H’ play an important role in the energy partitioning. Actually, the boundary waves excited from channel 1 not only can propagate to terminals 2 and 3, but also may transport to terminal 4 with spin flipping, because of spin non-conservation. However, the spin flipping effect can be neglected here. For the H-shaped sample in Supplementary Fig. 8b, the boundary states are decoupled between the top and bottom boundaries of the middle ribbon. While for the sample in Supplementary Figs. 8c and 8d, the spin flipping is too weak to be revealed, since operated frequency is in the band gap and the waves damp too

much along the lengthy middle ribbon ($L_{\mathrm{m}} = 13a$). To make the spin flipping observable, the H-shaped sample should be fabricated for a shorter $L_{\mathrm{m}} = 2a$, and the working frequency is tuned to the passing band, as discussed in Fig. 5 of the Main Text.

Supplementary Note 9. The projected dispersions of the middle ribbon in the H-shaped PC

In this section, we calculate the projected dispersions of the middle ribbon in the H-shaped PC. The results are shown in Supplementary Figs. 9a-9f for different widths of the bridge W_{m}. The red circle denotes the lowest frequency f_{b}^{l} of the up branch of the boundary states in the lower gap, while the blue square represents the highest frequency f_{b}^{h} of the down branch. These points are shown in the middle panel of Supplementary Fig. 8a. The green dashed line indicates the excitation frequency 7.44 kHz of the wave launched at channel 1 shown in Supplementary Fig. 8a. When $W_{\mathrm{m}} = 5.5a$, the gap of boundary waves is still completely closed, as shown in Supplementary Fig. 8a. For $W_{\mathrm{m}} < 5.5a$, shown in Supplementary Figs. 9b-9f, the gap of boundary state opens, due to the finite size effect. The smaller W_{m}, the stronger is the coupling between the upstream and downstream boundary waves. In turns, the size of the gap of the boundary modes is proportional to this coupling. Hence, changing W_{m} is an effective way to control the boundary gap and enables to efficiently control the surface waves.

Supplementary Note 10. The fragile topology in a similar structure

Very recently, a similar acoustic structure has been employed to provide the first experimental observation of a fragile topological insulator [8]. Although based on similar experimental setup, the two works tell completely different topological physics, this one regarding the spin-Chern insulator and that one regarding fragile topology [8].

It has been found that most of the reported topological bands in classical wave systems have fragile topology from the crystalline symmetry standpoint [8-10]. Fragile topology indicates the lack of stability under the addition of trivial bands to the system. While this idea is rather generic, it has prominent importance in the context of

crystalline topological insulators. A powerful framework to study the presence of fragile topology is offered by Topological Quantum Chemistry [11, 12], where all the possible trivial atomic insulators in the presence of constraining crystalline symmetries are listed. The fragile topology in Ref. [8] lies its foundation in this framework, and strongly hinges on the presence of C_2 symmetry and result in twisted bulk-boundary correspondence. Once C_2 symmetry is broken, the topological invariant (i.e. the real-space index) is ill-defined and the required twisted boundary for hosting the boundary states cannot be constructed. However, the spin-Chern number is independent on crystalline symmetries, thus is still well-defined in the absence of C_2 symmetry. The nonzero spin-Chern number results in the generalized bulk-boundary correspondence (S-III). As a concrete example, one can see that the system without C_2 symmetry still hosts the gapless helical edge states (Supplementary Figs. 5 and 6 in Supplementary Note 6).

Therefore, the fragile topology and the topology described by spin-Chern number are essentially different, although they can exist in a similar system.

Supplementary References

1. Weeks, C., Franz, M. Topological insulators on the Lieb and perovskite lattices. *Phys. Rev. B* **82**, 085310 (2010).
2. Fu, L., Kane, C. L. Topological insulators with inversion symmetry. *Phys. Rev. B* **76**, 045302 (2007).
3. Prodan, E. Robustness of the spin-Chern number. *Phys. Rev. B* **80**, 125327 (2009).
4. Li, H., Sheng, L., Sheng, D. N., Xing, D. Y. Chern number of thin films of the topological insulator Bi_2Se_3. *Phys. Rev. B* **82**, 165104 (2010).
5. Yang, Y. *et al.* Time-reversal-symmetry-broken quantum spin Hall effect. *Phys. Rev. Lett.* **107**, 066602 (2011).
6. Li, H. *et al.* Connection of Edge States to Bulk Topological Invariance in a Quantum Spin Hall State. *Phys. Rev. Lett.* **108**, 196806 (2012).
7. Zhang, L. B., Cheng, F., Zhai, F., Chang, K. Electrical switching of the edge channel transport in HgTe quantum wells with an inverted band structure. *Phys.*

Rev. B **83**, 081402 (2011).

8. Peri, V. *et al.* Experimental characterization of fragile topology in an acoustic metamaterial. *Science* **367**, 797-800 (2020).
9. Wang, H.-X. *et al.* Band topology in classical waves: Wilson-loop approach to topological numbers and fragile topology. *New J. Phys.* **21**, 093029 (2019).
10. Alexandradinata, A. *et al.* Crystallographic splitting theorem for band representations and fragile topological photonic crystals. Preprint at https://arxiv.org/abs/1908.08541 (2019).
11. Song, Z. *et al.* Twisted bulk-boundary correspondence of fragile topology. *Science* **367**, 794-797 (2020).
12. Bradlyn, B. *et al.* Topological quantum chemistry. *Nature* **547**, 298-305 (2017).
